# Burden of non-cancer comorbidities and mortality in chronic pancreatitis: a retrospective cohort study

**DOI:** 10.1136/bmjgast-2025-002194

**Published:** 2026-05-21

**Authors:** Shauntelle Quammie, Adil Rashid, Rahul Munyal, Edward S Nicholson, Christopher Clarke, Suresh V Venkatachalapathy, Colin John Crooks, Guruprasad P Aithal, Aloysious D Aravinthan, The DEMISTIFI Consortium, Andrew Thorley

**Affiliations:** 1 NIHR Nottingham Biomedical Research Centre, Nottingham University Hospitals NHS Trust and University of Nottingham, Nottingham, UK; 2 Nottingham Digestive Diseases Centre, Translational Medical Sciences, School of Medicine, University of Nottingham, Nottingham, UK; 3 Department of General Surgery, Northern General Hospital NHS Trust, Sheffield, UK; 4 Department of Radiology, Nottingham University Hospitals NHS Trust, Nottingham, UK; 5 Department of Pharmacy, Nottingham University Hospitals NHS Trust, Nottingham, UK

**Keywords:** CARDIOVASCULAR DISEASE, CHRONIC LIVER DISEASE, CHRONIC PANCREATITIS, DIABETES MELLITUS

## Abstract

**Objective:**

Chronic pancreatitis (CP) is a progressive fibro-inflammatory disease associated with increased comorbidity and mortality. This study aimed to characterise the natural history of CP by assessing comorbidity prevalence and mortality in a CP cohort.

**Methods:**

A retrospective cohort study was performed at Nottingham University Hospitals NHS Trust, including patients diagnosed with CP between 1 January 2006 and 31 December 2014. Participants were followed up until 31 May 2023. Comorbidity prevalence was compared with national data from England in 2022 to calculate standardised prevalence ratios (SPRs). Similarly, standardised mortality ratios (SMRs) were also evaluated.

**Results:**

Of the 1003 patients with CP, 678 who resided within the Greater Nottingham area were included in the study cohort. The overall median follow-up was 5.7 years (IQR 1.2–10.6). Compared with the general population, patients with CP had significantly elevated SPRs for heart failure (3.0; 95% CI 2.3 to 3.8), diabetes mellitus (2.4; 95% CI 2.1 to 2.7), ischaemic heart disease (2.4; 95% CI 2.0 to 2.8), atrial fibrillation (1.9; 95% CI 1.5 to 2.3) and cerebrovascular disease (1.4; 95% CI 1.0 to 1.8). The cumulative incidence at 20 years of diabetes mellitus, chronic liver disease and chronic kidney disease was 12%, 11% and 11%, respectively. During follow-up, 495 patients (73%) died, with median survival of 5.7 years (95% CI 4.8 to 6.4). The overall SMR of patients with CP was significantly increased (2.3; 95% CI 2.1 to 2.6; p<0.0001) compared with the general population. Leading causes of death were malignancy (27%), infections (22%) and cardiovascular disease (6.9%).

**Conclusion:**

CP is associated with increased risk of multiple comorbidities and elevated mortality as compared with the general population. Enhanced surveillance and early intervention strategies may help prevent or delay the onset of these associated conditions.

**WHAT IS ALREADY KNOWN ON THIS TOPIC**

- Chronic pancreatitis (CP) is associated with diabetes and bone disease, but its broader systemic comorbidity burden and contemporary mortality risk compared with the general population are poorly defined.

**WHAT THIS STUDY ADDS**

- In a large UK cohort, CP was associated with markedly increased risks of cardiovascular disease (including heart failure and ischaemic heart disease), cerebrovascular disease, chronic liver disease and diabetes, together with a two-fold increase in mortality, particularly from malignancy and infection.

**HOW THIS STUDY MIGHT AFFECT RESEARCH, PRACTICE OR POLICY**

- Routine cardiovascular and metabolic risk assessment should be incorporated into CP care pathways.
- Surveillance for liver disease and other high-risk comorbidities should be considered.
- Preventative strategies, including vaccination and early infection management, may reduce avoidable mortality.

## Introduction

 The global burden of chronic pancreatitis (CP) is rising, with a 20% increase in prevalence and 80% higher mortality over the past two to three decades.^1^,^3^ CP is a progressive fibroinflammatory disease affecting endocrine and exocrine function.^4^ Endocrine involvement leads to diabetes mellitus (DM) in 45%–90% of patients 20–50 years after diagnosis, establishing CP as a risk factor for DM.^5^,^7^

CP is also linked to osteopathy, with 25%–66% developing osteoporosis or osteopenia, likely due to malabsorption, chronic inflammation and other mechanisms.^8 9^ The prevalence of other comorbidities remains unclear, highlighting the need to assess whether CP independently increases risk of systemic conditions.^10 11^

Mortality in CP is poorly characterised, as studies are limited by small, outdated cohorts, inconsistent definitions and few centres.^12^,^15^ Further research is needed to define mortality patterns, explore screening for associated conditions and update knowledge on CP’s natural history.^14^,^19^

This study evaluates non-cancer comorbidities and mortality in a contemporary cohort of patients with CP.

## Methods

### Patient selection and data collection

A retrospective cohort study was conducted at Nottingham University Hospitals NHS Trust, a high-volume tertiary centre, to identify adult patients diagnosed with CP between 1 January 2006 and 31 December 2014. Case ascertainment was performed using multiple data sources, and CP diagnoses were confirmed according to established CT, MRI and endoscopic ultrasound criteria, as previously described.^20^ Data were extracted in May 2023 from electronic health records and supplemented by manual review of clinical notes to ensure accuracy. The end of follow-up was defined as 31 May 2023. The study is reported in accordance with Strengthening the Reporting of Observational Studies in Epidemiology (STROBE) guidelines; the completed STROBE checklist is provided in online supplemental material.

### Study cohort

The cohort included individuals diagnosed with CP who were identified using multiple data sources and were residents of the Greater Nottingham area. This geographical region was chosen to maximise the accuracy and completeness of follow-up and outcome data, as it falls predominantly within the catchment area of Nottingham University Hospitals NHS Trust (NUH).

### Comorbidities of interest and natural history

Non-cancer comorbidities were selected based on the 20 most prevalent conditions nationally, according to data from the Office for National Statistics (England) covering the period from March 2017 to March 2020. These included ischaemic heart disease (IHD), heart failure (HF), cerebrovascular disease (CVD), atrial fibrillation (AF), hypertension, chronic liver disease (CLD), DM, osteoporosis/osteopenia, chronic kidney disease (CKD) and dementia (including Alzheimer’s disease).^21^ All comorbidities were identified through International Classification of Diseases, 10th Revision coding and confirmed via electronic records. Surgical interventions related to complications of CP were also recorded as part of the disease burden. For conditions with fewer than five cases, values were recorded as ‘<5’ to preserve anonymity.

The prevalence of these comorbidities at the time of CP diagnosis was evaluated using standardised prevalence ratios (SPRs). Cumulative incidence of comorbidities developing after the diagnosis of CP, along with SPRs at the end of follow-up, was then assessed to characterise the natural history of comorbidity development in this patient cohort.

### Alcohol and smoking history

Alcohol exposure was defined as current or previous consumption >14 units/week. Any history of tobacco use was documented, with smoking burden recorded in pack-years.

### Mortality

Causes of death were obtained from electronic health records at NUH, based on the diagnoses listed on the death certificate. A subgroup analysis was conducted excluding patients who died within 18 weeks of their CP diagnosis, in order to explore potential causality by removing comorbidities that were diagnosed as part of the same tests performed to diagnose CP.

### Outcomes

The main aims were to evaluate the natural history of comorbidity development and to assess mortality in patients with CP. This included evaluating the prevalence and incidence of comorbidities (including surgical interventions related to CP), overall mortality and causes of death. These outcomes were selected to provide a comprehensive understanding of the long-term clinical course, disease burden and survival outcomes associated with CP.

### Statistical analysis and data presentation

Continuous variables are presented as median (IQR) and categorical variables as number (percentage), unless otherwise stated. Prevalence of each comorbidity in the study cohort was compared with age-stratified national data for England, sourced from the 2022 Health Surveys for hypertension and CKD, the 2023–2024 National Diabetes Audit and the 2022–2023 disease-specific registers for IHD, stroke/transient ischaemic attack, HF and AF. Prevalence of CLD was obtained from the Global Burden of Disease data (2022).

SPRs, adjusted for age and sex where possible, were calculated using the epiR package.^22^ Cumulative incidence was estimated using cmprsk and was adjusted for competing risk of death.^23^

Kaplan-Meier curves and median survival were generated using the survival package. The age-adjusted standardised mortality ratio (SMR) was calculated using popEpi^24^ comparing observed deaths in the CP cohort with the general population of England in 2022. A p value <0.05 was considered statistically significant. All analyses were conducted using R (V.4.3.3).

## Results

### Clinical characteristics

A total of 1003 patients with CP were identified, of whom 678 resided within the Greater Nottingham area and were included in the study cohort. The median age at diagnosis was 68 years (IQR 53–79). The majority of patients were male (n=450; 66.4%) and of European ancestry (n=625; 92.2%). The most common associated risk factors were smoking (n=366; 54.0%) and alcohol use (n=279; 41.2%) (table 1). The median follow-up was 5.7 years (IQR 1.2–10.6).

**Table 1:** Demographic and clinical characteristics of the study cohort (n=678)

Parameters	Median (IQR) or number (%)
Age at diagnosis (years)	68 (53–79)
Male sex	450 (66.4%)
History of smoking	366 (54.0%)
History of alcohol	279 (41.2%)
Ethnic origin	
European	625 (92.2%)
African	14 (2.1%)
Asian	8 (1.2%)
Mixed	<5 (<1%)
Unknown	29 (4.3%)
Comorbidities at diagnosis	
Hypertension	240 (35.4%)
DM	204 (30.1%)
IHD	146 (21.5%)
CKD	80 (11.8%)
Atrial fibrillation	79 (11.7%)
CLD	76 (11.2%)
Heart failure	63 (9.3%)
Cerebrovascular disease	53 (7.8%)
Osteoporosis/Osteopenia	50 (7.4%)
Dementia	44 (6.5%)

CKD, chronic kidney disease; CLD, chronic liver disease; DM, diabetes mellitus; IHD, ischaemic heart disease.

The most frequently observed comorbidities at the time of diagnosis of CP were hypertension (n=240; 35.4%), DM (n=204; 30.1%), IHD (n=146; 21.5%), depression (n=85; 12.5%), CKD (n=80; 11.8%), AF (n=79; 11.7%), CLD (n=76; 11.2%), HF (n=63; 9.3%), CVD (n=53; 7.8%) and osteoporosis/osteopenia (n=50; 7.4%) (table 1).

### Natural history of comorbidities

Compared with national data for England, patients with CP had significantly increased SPRs for HF (3.0 (95% CI 2.3 to 3.8); p<0.0001), for IHD (2.4 (95% CI 2.0 to 2.8); p<0.0001), for AF (1.9 (95% CI 1.5 to 2.3); p<0.0001), for CVD (1.4 (95% CI 1.0 to 1.8); p=0.02) and for DM (2.4 (95% CI 2.1 to 2.7); p<0.0001) at the time of CP diagnosis (table 2).

**Table 2:** Age-adjusted SPRs of comorbidities at CP diagnosis and at end of follow-up

Comorbidities	SPRs at CP diagnosis (95% CI)	P value	SPRs at end of follow-up (95% CI)	P value
Heart failure	3.0 (2.3 to 3.8)	<0.0001	3.8 (3.0 to 4.7)	<0.0001
DM	2.4 (2.1 to 2.7)	<0.0001	3.1 (2.8 to 3.5)	<0.0001
IHD	2.4 (2.0 to 2.8)	<0.0001	2.6 (2.2 to 3.0)	<0.0001
Atrial fibrillation	1.9 (1.5 to 2.3)	<0.0001	2.6 (2.2 to 3.2)	<0.0001
Cerebrovascular disease	1.4 (1.0 to 1.8)	0.02	2.5 (2.0 to 3.0)	<0.0001
CKD	0.7 (0.5 to 0.8)	<0.0001	1.1 (1.0 to 1.4)	0.1
CLD	0.7 (0.6 to 0.9)	0.004	1.4 (1.2 to 1.7)	<0.0001
Hypertension	0.7 (0.6 to 0.8)	<0.0001	0.9 (0.8 to 1.0)	0.009

CKD, chronic kidney disease; CLD, chronic liver disease; CP, chronic pancreatitis; DM, diabetes mellitus; IHD, ischaemic heart disease; SPR, standardised prevalence ratio.

The cumulative incidence of comorbidities developing 10 and 20 years after CP diagnosis was as follows: DM 8.3% and 12%, CLD 8.9% and 11%, CKD 7.2% and 11%, osteoporosis/osteopenia 6% and 8.8%, CVD 5.3% and 6.9%, hypertension 4.2% and 6.7%, AF 3.6% and 5.5%, dementia 2.1% and 3.8%, HF 1.7% and 2.5% and IHD 1.5% and 2.2% (table 3; online supplemental figure 1).

**Table 3:** Cumulative incidence of comorbidities after CP diagnosis

Comorbidities	Years since CP diagnosis			
	5	10	15	20
DM	6.1% (4.4 to 8.0)	8.3% (6.4 to 11.0)	10% (8.1 to 13.0)	12% (9.2 to 16.0)
CLD	6.1% (4.4 to 8.0)	8.9% (6.9 to 11.0)	9.9% (7.7 to 12.0)	11.0% (8.1 to 15.0)
CKD	4.6% (3.2 to 6.3)	7.2% (5.4 to 9.3)	8.6% (6.5 to 11.0)	11.0% (7.8 to 14.0)
Osteoporosis/Osteopenia	3.7% (2.5 to 5.3)	6.0% (4.3 to 7.9)	8.3% (6.2 to 11.0)	8.8% (6.5 to 12.0)
Cerebrovascular disease	3.0% (1.9 to 4.4)	5.3% (3.8 to 7.2)	6.9% (4.9 to 9.2)	6.9% (4.9 to 9.2)
Hypertension	1.9% (1.1 to 3.2)	4.2% (2.8 to 5.9)	5.7% (4.0 to 7.9)	6.7% (4.3 to 9.7)
Atrial fibrillation	1.5% (0.8 to 2.6)	3.6% (2.4 to 5.2)	5.0% (3.5 to 7.0)	5.5% (3.7 to 7.7)
Dementia	1.0% (0.5 to 2.0)	2.1% (1.2 to 3.4)	2.5% (1.5 to 4.0)	3.8% (1.6 to 7.5)
Heart failure	0.7% (0.3 to 1.6)	1.7% (0.9 to 2.9)	2.5% (1.4 to 4.2)	2.5% (1.4 to 4.2)
IHD	0.7% (0.3 to 1.7)	1.5% (0.8 to 2.6)	2.2% (1.1 to 3.8)	2.2% (1.1 to 3.8)

Values are expressed as cumulative incidence percentages with 95% CIs in parentheses.

CKD, chronic kidney disease; CLD, chronic liver disease; CP, chronic pancreatitis; DM, diabetes mellitus; IHD, ischaemic heart disease.

At the end of follow-up, patients with CP had significantly increased SPRs for HF (3.8 (95% CI 3.0 to 4.7); p<0.0001), for IHD (2.6 (95% CI 2.2 to 3.0); p<0.0001), for AF (2.6 (95% CI 2.2 to 3.2); p<0.0001), for CVD (2.5 (95% CI 2.0 to 3.0); p<0.0001), for DM (3.1 (95% CI 2.8 to 3.5); p<0.0001) and for CLD (1.4 (95% CI 1.2 to 1.7); p<0.0001) at the end of follow-up, compared with national data for England (table 2).

### Pancreatic surgery

A total of 40 patients (5.9%) underwent pancreatic surgery for complications related to CP, with a median time to surgery of 2.9 years (IQR 1.1–4.9) from diagnosis. The incidence rate of surgery for CP-related complications was 10 per 1000 person-years. The most common indication was intractable abdominal pain (n=17; 42.5%). The most frequently performed surgical procedure was distal pancreatectomy with splenectomy (n=17; 42.5%) (table 4).

**Table 4:** Indications for and types of pancreatic surgery in patients with chronic pancreatitis

Indications for pancreatic surgery	Total (n=40) Number (%)
Intractable abdominal pain	25 (62.5%)
Strictures (bile duct, duodenal stricture, pancreatic duct)	7 (17.5%)
Malignancy or suspicion of malignancy	<5
Abscess	<5
Unknown	<5

Types of pancreatic surgery	Total (n=40) Number (%)
Distal pancreatectomy and splenectomy	17 (42.5%)
Roux-en-Y lateral pancreaticojejunostomy	10 (25.0%)
Roux-en-Y hepaticojejunostomy	5 (12.5%)
Frey procedure	<5
Roux-en-Y enteroenterostomy with bypass gastrojejunostomy	<5
Pancreatectomy	<5

For indications and types of surgery involving fewer than five patients, the number was recorded as <5 to preserve anonymity and prevent potential identification patients.

### Mortality

Following a diagnosis of CP, 495 patients (73%) died during follow-up (online supplemental figure 2). Median survival from diagnosis was 5.7 years (95% CI 4.8 to 6.4). The leading causes of death were malignancy (n=134, 27.1%), infections (n=110, 22.2%) and cardiovascular disease (n=34, 6.9%) (table 5). The overall SMR for patients with CP was 2.3 (95% CI 2.1 to 2.6; p<0.0001) over 4588 person-years of follow-up, compared with the general population of England. The SMR was highest among those diagnosed at a younger age, declining gradually with increasing age at CP diagnosis (table 6).

**Table 5:** Primary causes of death in patients with chronic pancreatitis

Causes of death	Number (%)
Malignancy	134 (27.1%)
Lung	32
Pancreas	18
Oesophagus	13
Colorectal	13
Renal tract	10
Infection	110 (22.2%)
Pneumonia	80
Urosepsis	15
COVID-19 pneumonitis	6
Cholangitis/Biliary sepsis	5
**Cardiovascular disease**	34 (6.9%)
Congestive cardiac failure	15
Ischaemic heart disease	12
Liver disease—end-stage liver disease	24 (4.9%)
Acute abdomen	28 (5.7%)
Ischaemic colitis	9
Perforation	8
Respiratory	13 (2.6%)
COPD exacerbation	9
Others	151 (30.5%)
Unknown	110
Frailty	6

COPD, chronic obstructive pulmonary disease.

**Table 6:** Standardised Mortality Ratio (SMR) in the CP cohort compared with the population of England in 2022, by age category

Age category	Person years	SMR (95% CI)	P value
30-34	10.1	817.0 (265.3–1906.5)	<0.0001
35-39	56	178.8 (81.8–339.4)	<0.0001
40-44	203.5	38.6 (19.2–69.0)	<0.0001
45-49	299.3	23.5 (13.2–38.8)	<0.0001
50-54	301.8	19.2 (11.5–29.9)	<0.0001
55-59	415.2	12.2 (7.9–18.1)	<0.0001
60-64	509.3	10.2 (7.3–14.0)	<0.0001
65-69	367.8	10.2 (7.5–13.7)	<0.0001
70-74	489.8	6.6 (5.1–8.5)	<0.0001
75-79	568.8	3.3 (2.5–4.2)	<0.0001
80-84	474.3	2.8 (2.2–3.5)	<0.0001
85-89	507	1.6 (1.3–2.0)	<0.0001
90-99	384.9	0.5 (0.4–0.7)	<0.0001
Overall	4588	2.3 (2.1–2.6)	<0.0001

In a subgroup analysis excluding patients who died within 18 weeks of CP diagnosis, 575 patients were included. During follow-up, 391 (68%) died. Median survival in this subgroup was 7.3 years (95% CI 6.5 to 8.3). The leading causes of death were malignancy (n=100; 26%) and infection (n=89; 23%), consistent with the overall cohort. The overall SMR was 1.9 (95% CI 1.7 to 2.1; p<0.0001) over 4490 person-years of follow-up, compared with the general population of England. As in the overall cohort, the SMR was highest in those diagnosed at a younger age, with a progressive decline in SMR observed with increasing age at diagnosis (table 7).

**Table 6:** Standardised Mortality Ratio (SMR) in the CP cohort (excluding deaths within 18 weeks) compared with the population of England in 2022, by age category

Age category	Person years	SMR (95% CI)	P value
30-34	9.98	661.4 (180.2–1693.4)	<0.0001
35-39	55.62	140.0 (56.3–288.5)	<0.0001
40-44	119.12	65.9 (32.9–117.8)	<0.0001
45-49	299.1	20.4 (10.9–34.9)	<0.0001
50-54	301.49	16.1 (9.2–26.2)	<0.0001
55-59	414.2	8.8 (5.2–14.0)	<0.0001
60-64	507.94	8.1 (5.5–11.5)	<0.0001
65-69	367.01	8.0 (5.6–11.1)	<0.0001
70-74	488.26	5.3 (4.0–7.1)	<0.0001
75-79	567.35	2.6 (1.9–3.5)	<0.0001
80-84	472.46	2.2 (1.7–2.8)	<0.0001
85-89	503.65	1.2 (0.9–1.5)	0.3
90-99	384.29	0.5 (0.3–0.6)	<0.0001
Overall	4490	1.9 (1.7–2.1)	<0.0001

## Discussion

This study provides a comprehensive evaluation of the natural history of CP, focusing on the burden of non-cancer comorbidities and mortality within a large, contemporary cohort. Patients with CP exhibited significantly higher SPRs of cardiovascular comorbidities, including IHD, HF, AF and CVD, as well as CLD, CKD and DM, when compared with the general population of England adjusted for age. Importantly, CP was also associated with a twofold increased risk of death, largely driven by cancer and infections.

The association between CP and cardiovascular disease is biologically plausible and likely mediated by systemic inflammation. CP induces a pro-inflammatory state, characterised by elevated levels of cytokines such as tumour necrosis factor-α, interleukin-6 and interleukin-8, along with endothelial adhesion molecules like P-selectin and ICAM-1.^25^,^27^ These factors contribute to the formation and destabilisation of atherosclerotic plaques, which can precipitate cardiovascular events.^27^ This mechanism may also explain the increased SPRs for IHD, HF and CVD observed in this study, consistent with prior findings.^28^,^30^ Similarly, cigarette smoking and chronic excessive alcohol consumption increase cardiovascular disease risk by promoting endothelial and mitochondrial dysfunction, oxidative stress and the production of proinflammatory cytokines and chemokines.^31 32^ Smoking additionally induces a prothrombotic state, further contributing to cardiovascular disease development.^32^

A novel finding in this study was the potential link between CP and HF, possibly via pancreatic exocrine insufficiency. Prior work has demonstrated reduced faecal elastase levels in patients with HF, suggestive of subclinical pancreatic exocrine insufficiency, which improved with pancreatic enzyme replacement therapy.^33 34^ Reduced pancreatic perfusion in HF may partly explain this observation,^35^,^37^ raising the possibility that CP could occasionally be a sequela rather than a cause of HF.^33 37^ Similarly, this study confirmed an increased risk of AF in patients with CP, potentially driven by cardiac remodelling associated with chronic alcohol use and systemic inflammatory spillover from pancreatic inflammation.^38^,^41^

While alcohol is a well-established risk factor for both CP and CLD, only a minority of heavy drinkers develop either condition,^42^,^44^ suggesting the involvement of additional pathogenic mechanisms. CP may contribute to hepatic injury through pathways such as bacterial translocation, intestinal barrier disruption and cytokine-mediated hepatic fibrosis.^45 46^ In this study, the prevalence and cumulative incidence of CLD were significantly elevated in patients with CP, consistent with previous findings—most notably, a population-based study by Chand *et al*, which reported a 5-fold to 9-fold increased risk of cirrhosis and liver disease among individuals with CP.^47^

Diabetes is a well-established complication of CP, arising from chronic inflammation and progressive beta-cell dysfunction.^48^,^51^ Notably, although DM had the highest cumulative incidence of all comorbidities in this cohort, the observed rates (8.3% at 10 years; 12.0% at 20 years) were lower than previously reported figures of 15%–26% and up to 40% over comparable time intervals.^48 50^ This discrepancy may reflect competing mortality risks or represent a genuine regional variation in disease trajectory.

Surgical intervention for CP was required in only 6% of patients, considerably lower than historical estimates ranging from 40% to 75%.^52 53^ This lower rate may be attributable to the shorter follow-up period or increased reliance on minimally invasive or endoscopic therapies.^49^ Consistent with previous studies,^53^,^56^ the most common indication for surgery was intractable abdominal pain, with distal pancreatectomy and splenectomy being the most frequently performed procedure.

Mortality was substantial in this cohort, with 73% of patients dying over a median follow-up of 5.7 years. The leading causes of death were malignancy and infection. The age-adjusted SMR was more than twice that of the general population, aligning with existing literature.^12 15 57^ The increased susceptibility to infection and malignancy in CP may be explained by immune dysregulation. CP has been associated with impaired innate and adaptive immunity, including reduced numbers and functional activity of natural killer cells, cytotoxic T lymphocytes (CD8+) and CD25+ regulatory T cells.^58^,^62^ This immunosuppressed state may predispose patients to opportunistic infections. In parallel, chronic oxidative stress and inflammation, often exacerbated by alcohol use and smoking, are thought to contribute to carcinogenesis in CP.^63^ In this population, the median age at diagnosis of CP was higher than reported in previous studies, which may explain the greater proportion of deaths observed over a shorter period.

This study has strengths and limitations. It represents one of the largest single-centre cohorts of CP patients, systematically identified from a well-defined geographical region. Comprehensive case ascertainment strategies minimised selection bias,^20^ and data accuracy was enhanced through manual chart review and multiple-source validation. The use of national comparator data further allowed for robust benchmarking of comorbidity and mortality rates. However, certain limitations should be acknowledged. The retrospective design introduces potential for misclassification and missing data. The comparison with the general population was adjusted solely for age, as this was the only variable available. Furthermore, the CP cohort consisted predominantly of individuals of Caucasian descent, which limits the generalisability of the findings to other ethnic groups and populations. Although diagnoses were carefully verified and patients diagnosed only at death were excluded, diagnostic accuracy may still be affected by variability in documentation. To mitigate this, a sensitivity analysis excluding patients who died within 18 weeks of diagnosis was conducted and yielded consistent mortality outcomes. Additionally, under-reporting of alcohol and tobacco use may have introduced bias, potentially leading to an underestimation of the aetiological role of these factors in CP and their associated outcomes. Finally, the relatively short median follow-up and high mortality rate may have limited the ability to capture the full cumulative incidence of comorbidities with longer latency, such as DM and osteoporosis.

In conclusion, this study provides important insights into the long-term clinical trajectory of patients with CP. CP is associated with significantly increased risks of cardiovascular disease, diabetes, CLD and premature death, particularly from cancer and infection. These findings underscore the importance of routine screening for high-risk comorbidities and highlight the potential value of preventative strategies, including vaccination and early metabolic or cardiovascular intervention. Future prospective studies are needed to further delineate these relationships and identify opportunities for improved management of this high-risk population.

## Supplementary material

10.1136/bmjgast-2025-002194 online supplemental file 1

10.1136/bmjgast-2025-002194 online supplemental file 2

## Data Availability

All data relevant to the study are included in the article or uploaded as supplementary information.
