# Supplementary material for: Burden of non-cancer comorbidities and mortality in chronic pancreatitis: a retrospective cohort study
Source: BMJ Open Gastroenterol. 2026 May 21;13(1):e002194. doi: 10.1136/bmjgast-2025-002194 (PMC13202079; doi:10.1136/bmjgast-2025-002194)
Supplement: online supplemental file 1 [file bmjgast-13-1-s001.docx]

STROBE Statement—checklist of items that should be included in reports of observational studies

|  | Item No | Recommendation |
| --- | --- | --- |
| **Title and abstract** | 1 | (*a*) Indicate the study’s design with a commonly used term in the title or the abstract  Page 1, Page 3 line 8 |
|  |  | (*b*) Provide in the abstract an informative and balanced summary of what was done and what was found  Page 3 lines 7-24 |
| Introduction | | |
| Background/rationale | 2 | Explain the scientific background and rationale for the investigation being reported  Page 5 lines 46-66 |
| Objectives | 3 | State specific objectives, including any prespecified hypotheses  Page 5 lines 68-69 |
| Methods | | |
| Study design | 4 | Present key elements of study design early in the paper  Page 6, line 72 |
| Setting | 5 | Describe the setting, locations, and relevant dates, including periods of recruitment, exposure, follow-up, and data collection  Page 6 line 71-77 |
| Participants | 6 | (*a*) *Cohort study*—Give the eligibility criteria, and the sources and methods of selection of participants. Describe methods of follow-up  Page 6, lines 71-72, 82-83, 87-93  *Case-control study*—Give the eligibility criteria, and the sources and methods of case ascertainment and control selection. Give the rationale for the choice of cases and controls  *Cross-sectional study*—Give the eligibility criteria, and the sources and methods of selection of participants |
|  |  | (*b*) *Cohort study*—For matched studies, give matching criteria and number of exposed and unexposed  Not applicable  *Case-control study*—For matched studies, give matching criteria and the number of controls per case |
| Variables | 7 | Clearly define all outcomes, exposures, predictors, potential confounders, and effect modifiers. Give diagnostic criteria, if applicable  Outcomes – Page 7 , lines 114-115  Exposures – Page 7, lines 104-105 |
| Data sources/ measurement | 8* | For each variable of interest, give sources of data and details of methods of assessment (measurement). Describe comparability of assessment methods if there is more than one group  Sources of data: page 6, lines 87-93  Methods of assessment: page 6, lines 98-101, page 7, lines 120-135 |
| Bias | 9 | Describe any efforts to address potential sources of bias  Page 6, lines 109-111  Page 7, lines 129-130 |
| Study size | 10 | Explain how the study size was arrived at  Page 6, lines 72-74, 82-83 |
| Quantitative variables | 11 | Explain how quantitative variables were handled in the analyses. If applicable, describe which groupings were chosen and why  Page 7, line 121 |
| Statistical methods | 12 | (*a*) Describe all statistical methods, including those used to control for confounding  Page 7, lines 128-135 |
|  |  | (*b*) Describe any methods used to examine subgroups and interactions  Page 6 lines 109-111 |
|  |  | (*c*) Explain how missing data were addressed  The missing data was labelled as unknown. Page 16 line 432 table 1. Page 18 line 444 table 4. Page 19 line 451 table 5 |
|  |  | (*d*) *Cohort study*—If applicable, explain how loss to follow-up was addressed  Not applicable. Data from the GP practice regarding associated comorbidities was available to us.  *Case-control study*—If applicable, explain how matching of cases and controls was addressed  *Cross-sectional study*—If applicable, describe analytical methods taking account of sampling strategy |
|  |  | (*e*) Describe any sensitivity analyses |

Continued on next page

| Results | | |
| --- | --- | --- |
| Participants | 13* | 1. Report numbers of individuals at each stage of study—eg numbers potentially eligible, examined for eligibility, confirmed eligible, included in the study, completing follow-up, and analysed   This was detailed in another paper which was published in Frontline Gastroenterology and was referenced in this paper. Page 6, lines 74-75, Page 8, line 138-139 |
|  |  | 1. Give reasons for non-participation at each stage   This was detailed in another paper which was published in Frontline Gastroenterology and was referenced in this paper. Page 6, lines 74-75 |
|  |  | (c) Consider use of a flow diagram  This was detailed in another paper which was published in Frontline Gastroenterology and was referenced in this paper. Page 6, lines 74-75 |
| Descriptive data | 14* | (a) Give characteristics of study participants (eg demographic, clinical, social) and information on exposures and potential confounders  Page 8, line 139-141  Page 16, line 432, table 1 |
|  |  | (b) Indicate number of participants with missing data for each variable of interest  Data from the GP records was available to us. If the diagnosis/ comorbidity was not stated for the patient, then it was concluded that the patient did not have the diagnosis.  The only data missing were ethnicity which was stated as unknown in 29 (4.3%) of patients (page 16, line 432, table 1), indications for pancreatic surgery where <5, were stated as unknown (page 18, line 444, table 4) cause of death for some patients which were stated as unknown in 110 (page 19, line 451, table 5) |
|  |  | (c) *Cohort study*—Summarise follow-up time (eg, average and total amount)  Page 8, line 141 |
| Outcome data | 15* | *Cohort study*—Report numbers of outcome events or summary measures over time  Page 8, lines 143-146, lines 148-164, lines 167-169, 173-177 |
|  |  | *Case-control study—*Report numbers in each exposure category, or summary measures of exposure |
|  |  | *Cross-sectional study—*Report numbers of outcome events or summary measures |
| Main results | 16 | (*a*) Give unadjusted estimates and, if applicable, confounder-adjusted estimates and their precision (eg, 95% confidence interval). Make clear which confounders were adjusted for and why they were included  Page 8 lines 149 - 152, lines 160-164  We were adjusted for age in SPR and for competing risk of death in cumulative incidence |
|  |  | (*b*) Report category boundaries when continuous variables were categorized  Page 17, line 435, table 2  Page 18, line 440, table 3  Page 18, line 444, table 4  Page 19, line 451, table 5 |
|  |  | (*c*) If relevant, consider translating estimates of relative risk into absolute risk for a meaningful time period  Not applicable |
| Other analyses | 17 | Report other analyses done—eg analyses of subgroups and interactions, and sensitivity analyses  Page 9 lines 182-186 |
| Discussion | | |
| Key results | 18 | Summarise key results with reference to study objectives  Page 10, lines 190-195 |
| Limitations | 19 | Discuss limitations of the study, taking into account sources of potential bias or imprecision. Discuss both direction and magnitude of any potential bias  Page 11, lines 252-264 |
| Interpretation | 20 | Give a cautious overall interpretation of results considering objectives, limitations, multiplicity of analyses, results from similar studies, and other relevant evidence  Page 11, lines 271-275 |
| Generalisability | 21 | Discuss the generalisability (external validity) of the study results  Page 11, lines 257-261 |
| Other information | | |
| Funding | 22 | Give the source of funding and the role of the funders for the present study and, if applicable, for the original study on which the present article is based |

Page 1, Page 6 lines 74-75

*Give information separately for cases and controls in case-control studies and, if applicable, for exposed and unexposed groups in cohort and cross-sectional studies.

**Note:** An Explanation and Elaboration article discusses each checklist item and gives methodological background and published examples of transparent reporting. The STROBE checklist is best used in conjunction with this article (freely available on the Web sites of PLoS Medicine at http://www.plosmedicine.org/, Annals of Internal Medicine at http://www.annals.org/, and Epidemiology at http://www.epidem.com/). Information on the STROBE Initiative is available at www.strobe-statement.org.
