# Supplementary material for: Burden of non-cancer comorbidities and mortality in chronic pancreatitis: a retrospective cohort study
Source: BMJ Open Gastroenterol. 2026 May 21;13(1):e002194. doi: 10.1136/bmjgast-2025-002194 (PMC13202079; doi:10.1136/bmjgast-2025-002194)
Supplement: online supplemental file 2 [file bmjgast-13-1-s002.docx]

**SUPPLEMENTAL DATA (FIGURES AND TABLES)**

**Supplemental Figure 1: Cumulative incidence of comorbidities at 5, 10, 15 and 20 years following a diagnosis of Chronic Pancreatitis**


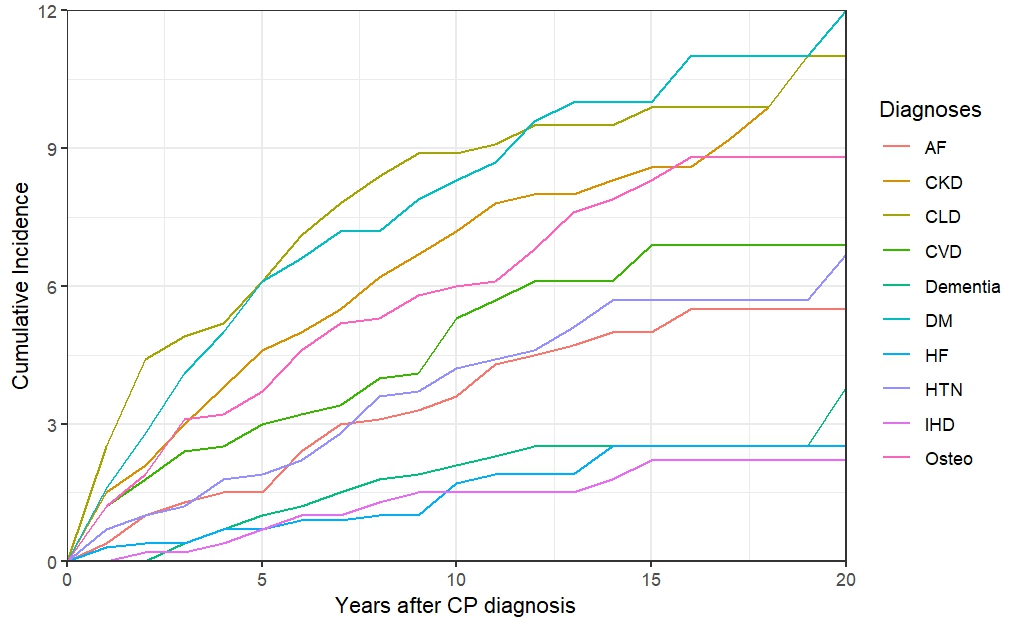


**Supplemental Figure 2: Kaplan–Meier curve illustrating overall survival probability following diagnosis of Chronic Pancreatitis.**


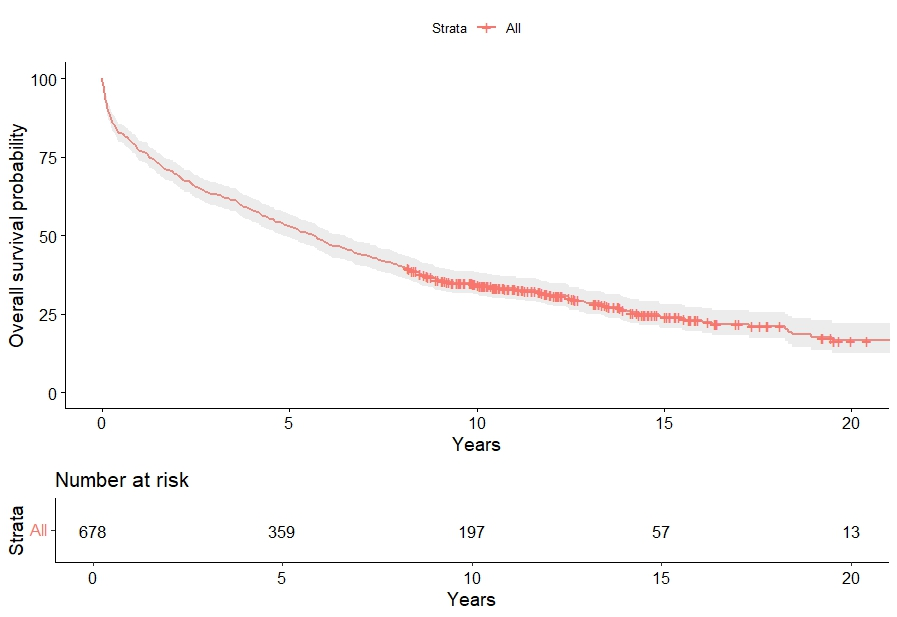


**Supplemental Table 1: Standardised Mortality Ratio (SMR) of CP cohort compared with the population of England**

| Supplemental Table 1A: Standardised Mortality Ratio (SMR) in the CP cohort compared with the population of England in 2022, by age category | | | |
| --- | --- | --- | --- |
| **Age Category** | **Person Years** | **SMR (95% CI)** | **p value** |
| 30-34 | 10.1 | 817.0 (265.3 – 1906.5) | <0.0001 |
| 35-39 | 56.0 | 178.8 (81.8- 339.4) | <0.0001 |
| 40-44 | 203.5 | 38.6 (19.2- 69.0) | <0.0001 |
| 45-49 | 299.3 | 23.5 (13.2-38.8) | <0.0001 |
| 50-54 | 301.8 | 19.2 (11.5-29.9) | <0.0001 |
| 55-59 | 415.2 | 12.2 (7.9-18.1) | <0.0001 |
| 60-64 | 509.3 | 10.2 (7.3-14.0) | <0.0001 |
| 65-69 | 367.8 | 10.2 (7.5-13.7) | <0.0001 |
| 70-74 | 489.8 | 6.6 (5.1-8.5) | <0.0001 |
| 75-79 | 568.8 | 3.3 (2.5-4.2) | <0.0001 |
| 80-84 | 474.3 | 2.8 (2.2-3.5) | <0.0001 |
| 85-89 | 507.0 | 1.6 (1.3-2.0) | <0.0001 |
| 90-99 | 384.9 | 0.5 (0.4-0.7) | <0.0001 |
| **Overall** | **4588** | **2.3 (2.1- 2.6)** | **<0.0001** |
|  |  |  |  |
|  |  |  |  |
| Supplemental Table 1B: Standardised Mortality Ratio (SMR) in the CP cohort (excluding deaths within 18 weeks) compared with the population of England in 2022, by age category | | | |
| **Age Category** | **Person Years** | **SMR (95% CI)** | **p value** |
| 30-34 | 9.98 | 661.4 (180.2 – 1693.4) | <0.0001 |
| 35-39 | 55.62 | 140.0 (56.3 – 288.5) | <0.0001 |
| 40-44 | 119.12 | 65.9 (32.9 – 117.8) | <0.0001 |
| 45-49 | 299.10 | 20.4 (10.9 – 34.9) | <0.0001 |
| 50-54 | 301.49 | 16.1 (9.2 – 26.2) | <0.0001 |
| 55-59 | 414.20 | 8.8 (5.2 – 14.0) | <0.0001 |
| 60-64 | 507.94 | 8.1 (5.5 – 11.5) | <0.0001 |
| 65-69 | 367.01 | 8.0 (5.6 – 11.1) | <0.0001 |
| 70-74 | 488.26 | 5.3 (4.0 – 7.1) | <0.0001 |
| 75-79 | 567.35 | 2.6 (1.9 – 3.5) | <0.0001 |
| 80-84 | 472.46 | 2.2 (1.7 – 2.8) | <0.0001 |
| 85-89 | 503.65 | 1.2 (0.9 – 1.5) | 0.3 |
| 90-99 | 384.29 | 0.5 (0.3 – 0.6) | <0.0001 |
| **Overall** | **4490.0** | **1.9 (1.7 – 2.1)** | **<0.0001** |
